# Transcriptome Analysis Reveals the Multiple Functions of pBD2 in IPEC-J2 Cells against *E. coli*

**DOI:** 10.3390/ijms23179754

**Published:** 2022-08-28

**Authors:** Shaoqiang Lian, Xiaqing Lin, Fengting Zhan, Xiaoyang Shen, Yu Liang, Chunli Li

**Affiliations:** 1 College of Animal Science and Technology, Henan Agricultural University, Zhengzhou 450046, China; 2 School of Medicine, Southern University of Science and Technology, Shenzhen 518055, China

**Keywords:** defensins, transcriptome analysis, *E. coli* infection, functions, immune response

## Abstract

Defensins play an important role in fighting bacteria, and are a good candidate for bactericidal agents. However, the function and mechanism of defensins in regulating host responses against bacteria is unclear. In this study, transcriptome analysis was used to study the comprehensive functions of pBD2 in IPEC-J2 cells against *E. coli*. In total, 230 differentially expressed genes (DEGs) were identified in IPEC-J2 cells between the control and *E. coli* groups, and were found by KEGG analysis to be involved in many signaling pathways related to immunity. Furthermore, 812 DEGs were observed between *E. coli* and *E. coli* +pBD2 groups, involved in the ribosome, oxidative phosphorylation, and certain disease pathways. Among these, 94 overlapping DEGs were in the two DEG groups, and 85 DEGs were reverse expression, which is involved in microRNA in cancer, while PTEN and CDC6 were key genes according to PPI net analysis. The results of qRT-PCR verified those of RNA-seq. The results indicated that pBD2 plays an important role against *E. coli* by acting on the genes related to immune response, cell cycle, ribosomes, oxidative phosphorylation, etc. The results provide new insights into the potential function and mechanism of pBD2 against *E. coli*. Meanwhile, this study provides a certain theoretical basis for research and the development of novel peptide drugs.

## 1. Introduction

In the past, the long-term use and abuse of antibiotics in animal husbandry has resulted in serious antibiotic residues and increase of drug-resistant strains, which is currently considered a great threat to public health [1]. Although the use of antibiotics in livestock has been limited as much as possible in recent years, this has brought some other challenges, such as the increase of intestinal diseases and the weakening of growth performance, especially at the weaning stage in the pig industry. It is urgent to develop new antimicrobial agents; host defensins may be one of the methods to solve this problem [2].

Defensins, members of the antimicrobial peptide family, exist widely in nature. They have shown bactericidal activity against gram-positive bacteria, gram-negative bacteria, and even multidrug-resistant bacteria; moreover, they have unique antibacterial mechanisms which make it difficult to induce bacterial resistance [3]. Defensins are part of the innate defense system and play an important role in immune response [2,4,5]. In addition, defensins could regulate the proliferation and migration of epithelial cells, which is very important for overcoming injury, infection and inflammation [4]. These advantages of defensins make them ideal antibiotic substitutes.

Porcine beta defensin 2 (pBD2) is an antibacterial peptide secreted by pigs. pBD2 has shown good antibacterial activity and immunomodulatory function in vitro [6,7,8,9]. pBD2 could inhibit *E. coli* and *S. aureus*, as well as some clinically isolated multidrug-resistant bacteria [9]. pBD2 has the strongest antimicrobial activity among the porcine beta defensins (pBDs) detected, including pBD1, pBD2, pBD114, and pBD129 [9,10,11,12]. In addition, pBD2 could alleviate the inflammatory response induced by exogenous stimulation in mice [8]. As a feed additive, pBD2 showed a certain effect on disease resistance and growth promotion, related to the reduction of numbers of harmful bacteria [13]. pBD2 may be a suitable antibiotic substitutes in the pig industry. However, the function and mechanism of how pBD2 protects the host from bacteria remains unclear.

In order to explain the functions and mechanism of pBD2 in the host cells against bacteria, *Escherichia coli* was selected as an example and intestinal porcine enterocyte cells (IPEC-J2) were selected as the infected model. *E. coli* is a gram-negative bacteria that exists widely in the intestinal tracts of animals. It is one of the most important pathogenic bacteria causing diarrhea in weaned piglets, which leads to considerable economic losses [14]. The IPEC-J2 cell line was originally isolated from the jejunum of a neonatal unsuckled piglet, and is a non-transformed, permanent intestinal cell line. IPEC-J2 cells are ideal for studying the antibacterial effect of pBD2 on cells [15]. In this experiment, IPEC-J2 cells were challenged with *E. coli*, and the transcriptome method was used to detect the effect of pBD2 on the gene expression of cells infected by *E. coli*. Our study helps to understand the functions and mechanisms of defensin in host cells against *E. coli*.

## 2. Results

### 2.1. Analysis of Bactericidal Activity

**Figure 1:**
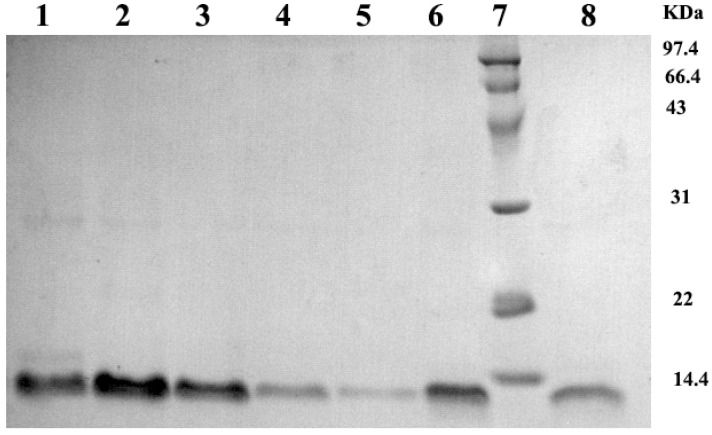
Analysis of purified pBD2 by SDS-PAGE. Lanes 1, 2, 3, 4, 5, 6, 8 indicate the purified protein from different collected tubes; Lane 7 indicates the protein marker.

**Figure 2:**
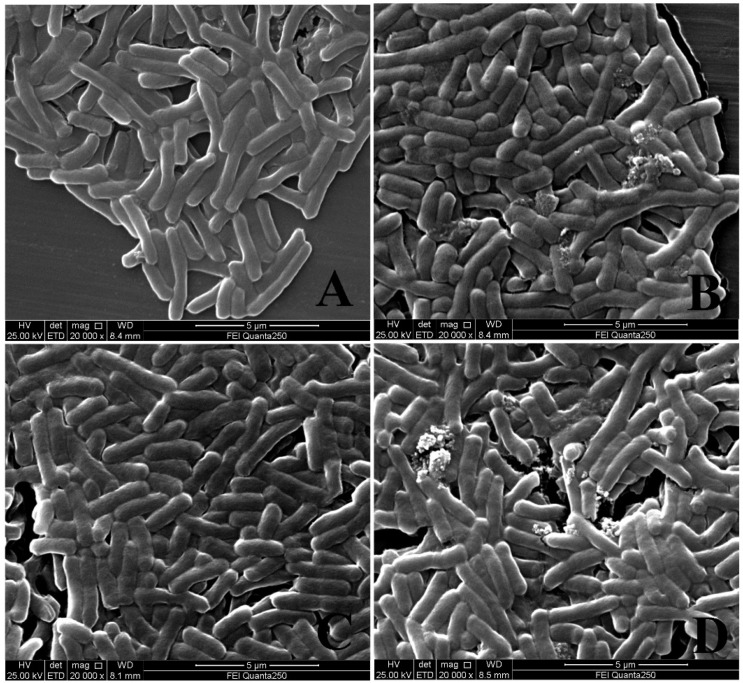
Morphological changes of *E. coli* treated with pBD2, viewed by scanning electron microscopy. (**A**,**B**) indicate the *E. coli* treated with 0 and 20 μg/mL pBD2 for 1 h, respectively; (**C**,**D**) indicated the *E. coli* treated with 0 and 20 μg/mL pBD2 for 4 h, respectively.

The recombinant pBD2 was induced and purified following our previous study [9]. The purified pBD2 showed high purity, and the molecular weight was approximately 12 kDα as expected (Figure 1). The recombinant pBD2 showed high bactericidal activity. *E. coli* were cracked and dead after incubation with 20 μg/mL pBD2 for 1 h and 4 h, the debris was observed, and the bactericidal effect was more obvious at 4 h than those at 1 h (Figure 2).

### 2.2. Transcriptome Profiling

cDNA libraries were sequenced on the Illumina high-throughput platform, generating significant amounts of high-quality raw data. After removal of adaptor sequences, low-quality, and contaminated reads, only the resulting clean reads were assembled to build transcripts. In these results, 20,721,673–35,369,943 clean data were obtained for samples, and the minimum Q30 was 93.23% (Table 1). The percentage of reads mapped to the reference genome was 95.84–96.55% (Table 2).

### 2.3. Characterization of Differentially Expressed Genes by RNA Sequencing

There were 230 differential expressed genes (DEGs) between the control and *E. coli* treatments, including 145 significantly upregulated genes and 85 significantly downregulated genes. There were 812 DEGs were between the *E. coli* and *E. coli* +pBD2 groups, including 431 significantly upregulated genes and 381 significantly downregulated genes. There were 94 overlapping genes observed by Venn diagram analysis between the two DEG groups.

### 2.4. KEGG Analysis Revealed That Immune Responses of Cells Were Trigger by E. coli

**Figure 3:**
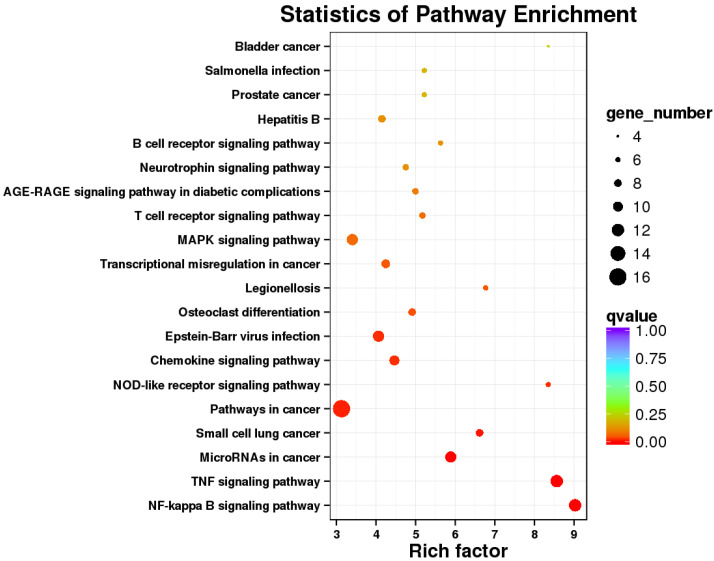
KEGG pathway analysis of DEGs between the control and *E. coli* groups.

**Figure 4:**
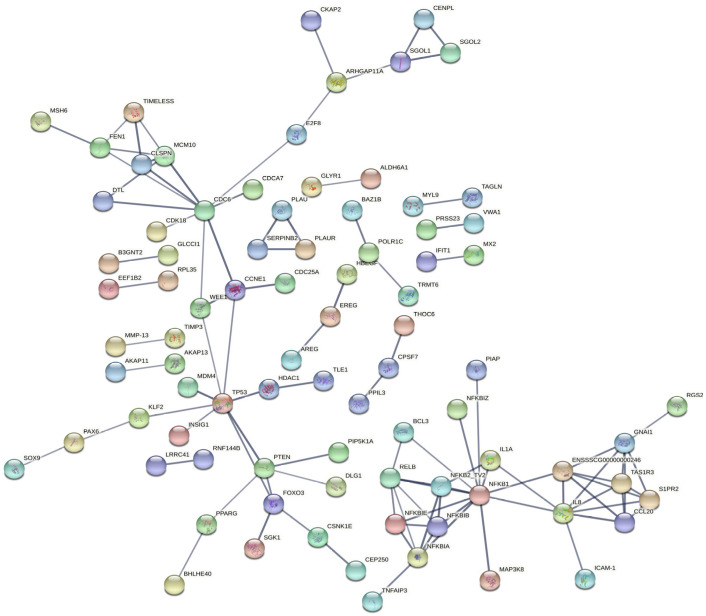
PPI analysis of DEGs in the control and *E. coli* groups.

To determine the functions of the DEGs involved, Kyoto Encyclopedia of Genes and Genomes (KEGG) annotation and corresponding enrichment analysis were performed. KEGG pathway analysis of the DEGs between the *E. coli* and control groups revealed that the immune pathways were significantly enriched, including NF-kappa B signaling pathway, TNF signaling pathway, NOD-like receptor signaling pathway, Chemokine signaling pathway, MAPK signaling pathway, etc., suggesting that *E. coli* caused immune responses of IPEC-J2 cells to eliminate the invading microbes (Figure 3). These results clearly indicate that the transcriptome of IPEC-J2 cells was changed after infection. Genes involved in the immune signaling pathways are listed in Table 3. The genes related to the immune responses include CXCL2 (chemokine (C-X-C motif) ligand 2), PTGS2 (prostaglandin G/H synthase 2), TNFAIP2 (tumor necrosis factor alpha-induced protein 2), NFKB2 (nuclear factor NF-kappa-B p100 subunit), NFKBIZ (NF-kappa-B inhibitor zeta), IL11 (interleukin-11), CCL20 (C-C motif chemokine ligand 20), etc. According to the PPI (protein–protein interaction) net analysis of the 230 DEGs, NFKB1(Nuclear factor NF-kappa-B p105 subunit), TP53 (tumor protein p53), PTEN (Phosphatidylinositol 3,4,5-trisphosphate 3-phosphatase) and CDC6 (cell division control protein 6) may be the key genes in IPEC-J2 cells in response to *E. coli* (Figure 4).

### 2.5. KEGG Pathway Analysis Revealed That pBD2 Has Multiple Functions in IEPC-J2 Cells against E. coli

**Figure 5:**
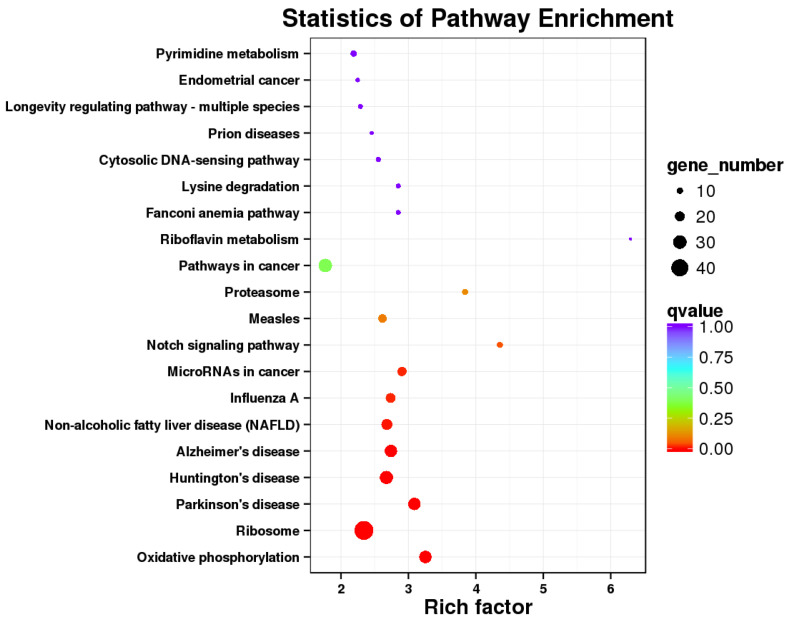
KEGG pathway analysis of DEGs in the *E. coli* and *E. coli* +pBD2 groups.

**Figure 6:**
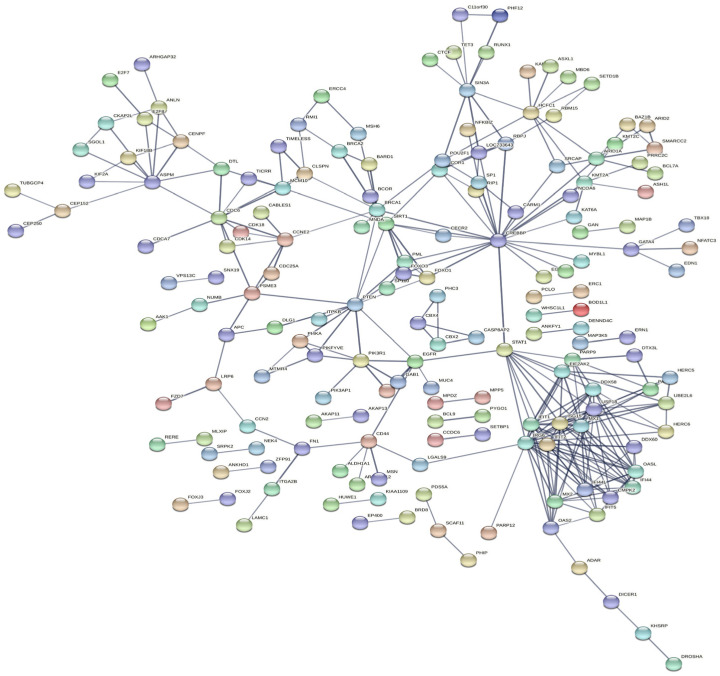
PPI analysis of the upregulated DEGs in the *E. coli* and *E. coli* +pBD2 groups.

**Figure 7:**
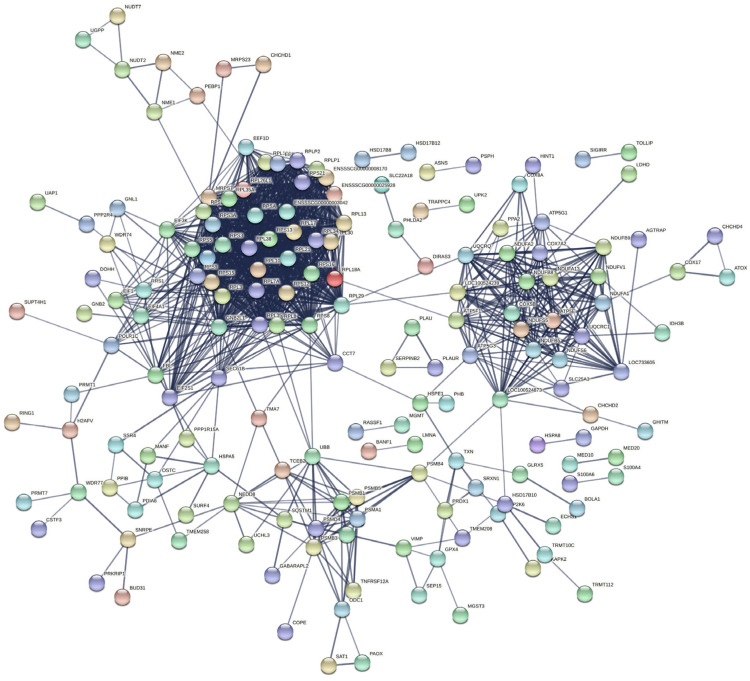
PPI analysis of the downregulated DEGs in the *E. coli* and *E. coli* +pBD2 groups.

There were 812 DEGs between the *E. coli* and *E. coli* +pBD2 groups. KEGG pathway analysis revealed DEGs involved in ribosomes, oxidative phosphorylation, and some diseases including Parkinson’s disease, Huntington’s disease, Alzheimer’s disease, Non-alcoholic fatty liver disease (NAFLD), and others (Figure 5). These results clearly indicate that the transcriptome of IPEC-J2 cells was regulated by pBD2. The 431 upregulated DEGs and 381 downregulated DEGs were analyzed by PPI net, respectively (Figure 6 and Figure 7). The results showed that the DEGs related to immune responses were obvious in the upregulated DEGs by PPI analysis, including MX1 (interferon-induced GTP-binding protein Mx1), EIF2AK2 (eukaryotic translation initiation factor 2 alpha kinase 2), OAS2 (2′-5′-oligoadenylate synthetase 2), IFIT1 (interferon-induced protein with tetratricopeptide repeats 3), IFIT2 (interferon-induced protein with tetratricopeptide repeats 2), IFIT5 (interferon-induced protein with tetratricopeptide repeats 5), IF144 (interferon-induced protein 44), etc. STAT1 (signal transducer and activator of transcription 1) and CREBBP (CREB binding protein) were the key genes (Figure 6). The downregulated DEGs were clustered into three groups (ribosome, oxidative phosphorylation, and proteasome) (Figure 7). These results indicate that pBD2 has multiple functions in cells against *E. coli*.

### 2.6. Comparison of pBD2 Effect with E. coli-Induced Transcriptome Changes

**Figure 8:**
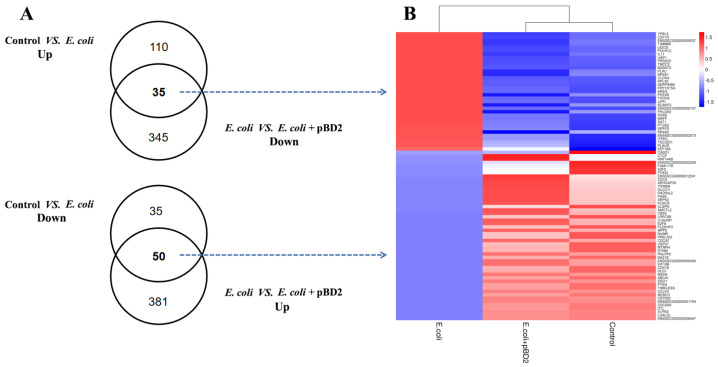
Overview of the DEGs after different treatments. (**A**) Venn diagrams of known DEGs based on comparisons of different groups. (**B**) Hierarchical clustering of the overlapping DEGs among different groups. The red indicates upregulated genes and the blue indicates downregulated genes.

There were 94 overlapping DEGs by Venn diagram analysis, which enabled us to identify transcripts specifically regulated by pBD2. Among these 94 DEGs, 85 DEGs were reverse expressed including 35 DEGs upregulated by *E. coli* and downregulated by pBD2 under *E. coli* stimulation (*E. coli* + pBD2 group). The other 50 DEGs were reverse expressed conversely to the above genes in both DEG groups (Figure 8).

**Figure 9:**
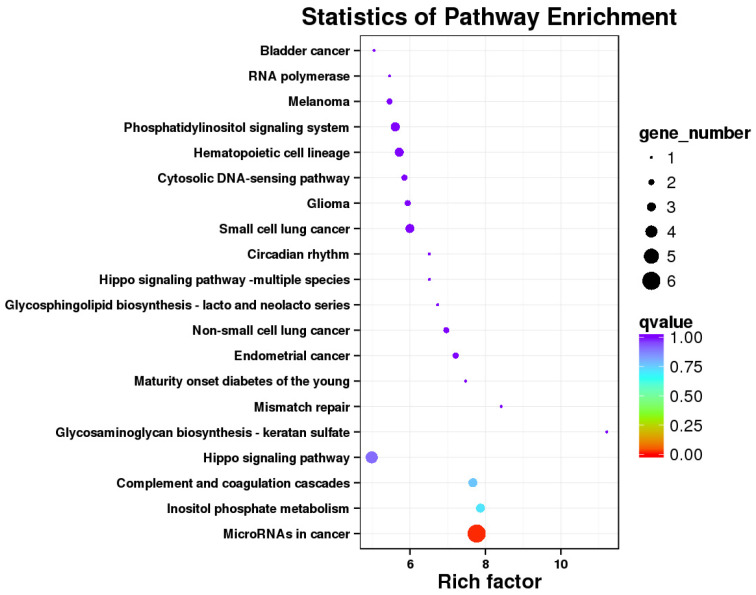
KEGG enrichment analysis of the DEGs overlapping between the two DEGs groups.

**Figure 10:**
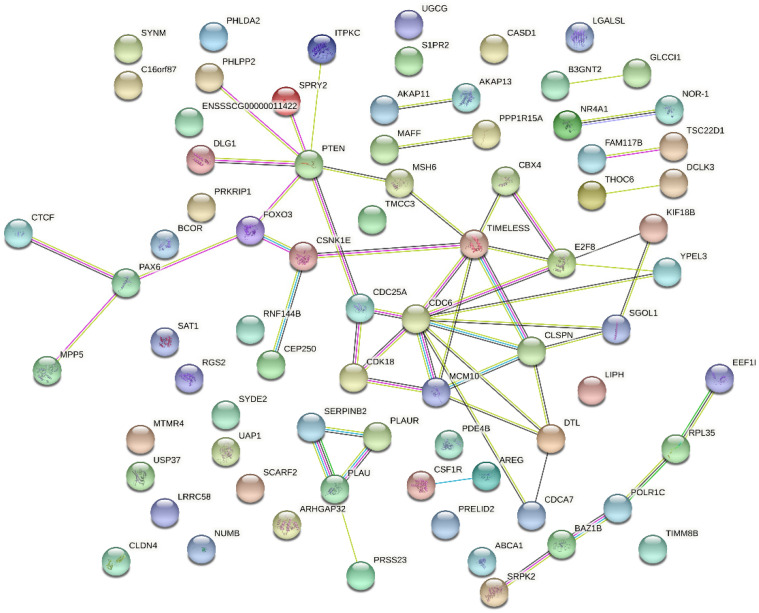
PPI analysis of the DEGs overlapping between the two DEG groups.

The KEGG pathway analysis of 85 DEGs with reverse expression showed that microRNA in cancer was the only significantly different signaling pathway, including PTEN, CDC25A (cell division cycle 25A), SPRY2 (protein sprouty homolog 2), E2F2 (E2F2 transcription factor), PTGS2, and PLAU (urokinase-type plasminogen activator) (Figure 9). In addition, PTEN and CDC6 were the hub genes in the PPI network for pBD2 to regulate IPEC-J2 cell responses to *E. coli* (Figure 10). In addition, immune-response-related genes among the 85 reverse expressed genes were considered. The immune-response-related genes IL11, PTGS2 and PLAU were all upregulated in the *E. coli* group compared with the control group, and pBD2 significantly downregulated the expression of these genes compared with the *E. coli* group.

### 2.7. Validation of RNA-seq Data by qRT-PCR

**Figure 11:**
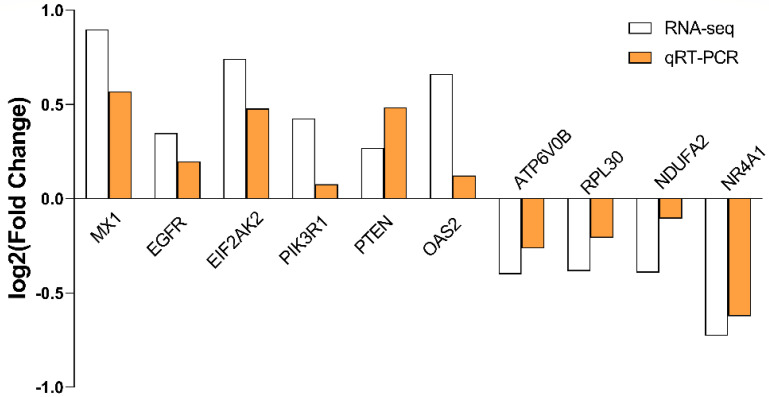
The verification of RNA-Seq results by qRT-PCR, between *E. coli* and *E. coli* +pBD2 groups. The samples were analyzed in triplicate by qRT-PCR, and fold-changes in gene expression were calculated by 2^−^^ΔΔCT^ methods with TUBA1B (tubulin alpha 1b) as a reference gene. MX1: Interferon-induced GTP-binding protein Mx1; EGFR: Epidermal growth factor receptor; EIF2AK2: Eukaryotic translation initiation factor 2 alpha kinase 2; PIK3R1: Phosphoinositide-3-kinase regulatory subunit alpha; PTEN: Phosphatidylinositol 3,4,5-trisphosphate 3-phosphatase; OAS2: 2′-5′-oligoadenylate synthetase 2; ATP6V0B: ATPase H+ transporting V0 subunit b; RPL30: Ribosomal protein L30; NDUFA2: NADH dehydrogenase [ubiquinone] 1 alpha subcomplex subunit 2; NR4A1: Nuclear receptor subfamily 4, group A, member 1.

Ten genes were selected for validation of RNA-seq, and the results of qRT-PCR were consistent with those of transcriptomics (Figure 11).

## 3. Discussion

Defensins, a kind of antimicrobial peptide, play an active role in resisting the invasion of pathogenic microorganisms [16]. pBD2 is one of the porcine beta defensins and shows strong antimicrobial activity against *E. coli*, *S. aureus* and isolated multidrug-resistant bacteria, with the advantages of high salt-tolerance, thermal stability, and low hemolytic activity, as demonstrated in our previous studies [9]. In addition, pBD2 has an immunomodulatory function [8]. However, the function and mechanisms of defensins in protecting the host from bacteria are still unclear.

In this study, we explored by transcriptomic analyses the effects of defensins on IPEC-J2 cells against *E. coli*. A total of 230 DEGs were obtained between the control and *E. coli* groups. The significant signaling pathways were mainly involved in immune responses, including NF-κB signaling pathway, TNF signaling pathway, NOD-like receptor signaling pathway, chemokine signaling pathway, etc., as demonstrated by KEGG enrichment analysis. The DEGs in these signaling pathways were upregulated, including NFKB1, NFKB2, NFKBIA (NF-kappa-B inhibitor alpha), NFKBIB (NF-kappa-B inhibitor beta), cytokines CXCL2, CCL20, IL1α (Interleukin 1 alpha), CXCL8 (C-X-C motif chemokine ligand 8), etc. Furthermore, NFKB1, TP53, PTEN, and CDC6 were key genes against *E. coli*, identified in the cells by PPI net analysis. NFKB1 is a key gene in the NF-κB signaling pathway, and PTEN is an important gene in the PI3K phosphatidyl inositol 3-kinase (PI3K)–protein kinase B (PKB/Akt) signaling pathway, which is involved in cell proliferation, differentiation, apoptosis, and immunity [17]. TP53 and PTEN are essential for initiating apoptosis and inflammatory response [18,19]. NFKB1, TP53, and PTEN were all upregulated, and were closely related to inflammatory response. The transcriptomic analyses indicated that immune responses of IPEC-J2 cells were triggered by *E. coli*, by which cells could eliminate invading pathogens for survival. It has been reported that multiple signaling pathways related to inflammation were activated by *E. coli* or LPS, resulting in the release of inflammatory cytokines [20,21,22,23,24,25]; those results were consistent with ours. CDC6 was a key gene for cell proliferation by PPI net analysis (Figure 4). In addition to CDC6, CDK18 (cyclin-dependent kinase 18), E2F8 (E2F8 transcription factor), CDCA7 (cell division cycle-associated protein 7), MCM10 (minichromosome maintenance 10 replication initiation factor) and TIMELESS (protein timeless homolog) are all related to cell proliferation according to their functions (Figure 4). It has been reported that CDC6 and CDK18 promoted proliferation of epithelial cells and inhibited apoptosis [26]. These genes were all decreased in the *E. coli* group, which perhaps suggest that cell proliferation was inhibited by *E. coli*.

There were 812 DEGs between the *E. coli* and *E. coli* +pBD2 groups, including 431 upregulated and 381 downregulated genes. PPI net analysis confirmed that the genes in the upregulated DEGs related to immune responses and cell proliferation, and STAT1 and CREBBP were the key genes (Figure 6). These genes are involved in many signaling pathways, including JAK-STAT and PI3K-AKT, which are related to immune responses. Our results were consistent with previous findings that defensin regulates immune responses in complex ways [27]. The genes related to cell proliferation in the upregulated DEGs included CDC6, CDK18, CDK14, E2F8, CDCA7, MCM10, TIMELESS, CDC25A, CCNE2 (G1/S-specific cyclin-E2), etc., according to their functions and the PPI net analysis. These genes were all increased by pBD2, which indicates that pBD2 protected cells from *E. coli* infection by regulating cell proliferation and inhibiting apoptosis, which might be critical for the resolution of injury, infection, and inflammation [2]. pBD2 upregulated DEGs related to immune responses and cell proliferation, which perhaps indicates that pBD2 could promote cell proliferation and inhibit the immune responses caused by *E. coli*.

The downregulated DEGs were obviously clustered into three groups (ribosome, oxidative phosphorylation, and proteasome) (Figure 7), which indicates that pBD2 had multiple functions in the cells. The ribosome is a complex molecular machine composed of numerous distinct proteins and nucleic acids, and is responsible for the translation of information contained in mRNAs into functional proteins, which play an important role in the execution of gene-expression programs, regulating basic biological processes such as cell growth, cell division, and differentiation. The hyperactivation of ribosome biogenesis has a critical role in cancer initiation and progression [28], and inhibition of ribosome biogenesis represents a potential therapeutic avenue for cancer treatment [29]. pBD2 downregulated the DEGs in the ribosome, perhaps implying that pBD2 could play a role in inhibiting the occurrence of cancer. The DEGs in oxidative phosphorylation (OXPHOS) were related to many diseases, and were also downregulated. OXPHOS was another source of ATP in cells in addition to glycolysis. Although it was reported that OXPHOS is related to cancer, many highly proliferative cell types including many cancer cells can preferentially utilize glycolysis [30,31,32,33]. In addition, OXPHOS inhibitors have heralded novel uses either for treating cancers in which OXPHOS is upregulated or alleviating tumor hypoxia to improve treatment outcomes [34]. In this study, pBD2 could downregulate the DEGs related to OXPHOS, which perhaps further implies that pBD2 could treat cancer. It was reported that peptide antibiotic leucinostatins showed inhibitory action on OXPHOS [35], which is consistent with our results. It was reported that the DEGs in OXPHOS were significantly enriched and upregulated in the large yellow croaker (*Larimichthys crocea*) when infected by *Pseudomonas* *plecoglossicida,* and were also upregulated in the murine model when infected by *R. conorii* [36,37]. These findings suggest that OXPHOS was related to immune responses and contributed to anti-infection strategies in the hosts. The mechanism underlying this strengthened energy metabolism is unknown, and the DEGs in OXPHOS were not enriched between the control and *E. coli* infection groups in this study. pBD2 could downregulate the DEGs, perhaps implying that pBD2 could protect the host from excessive inflammatory responses.

The proteasome is a large protein complex, responsible for the degradation of intracellular proteins, which regulates cellular proteostasis through selective degradation of ubiquitylated proteins [38,39]. Thereby, it performs a crucial role in cellular regulation and homeostasis, and has an important function in a variety of basic cellular processes including regulation of cell cycle progression, signal transduction, modulation of immune and inflammatory responses, etc. Malfunction of the ubiquitin-proteasome system (UPS) contributes to various diseases including cancer, inflammation, and neurodegeneration [40]. In this study, pBD2 inhibited the DEGs in the proteasome, and even lowered some ubiquitin expression including UBB (polyubiquitin-B), NEDD8, UBE2J2 (ubiquitin-conjugating enzyme E2 J2), APC2 and APC11 (anaphase-promoting complex subunit 2 and 11), proteasomal ubiquitin receptor UCHL3 (ubiquitin carboxyl-terminal hydrolase isozyme L3), USP19 (ubiquitin carboxyl-terminal hydrolase 19) genes, etc., all perhaps further indicating that pBD2 inhibited inflammations and the occurrence of cancer, as discussed above. It was reported that PR39, one of the porcine antimicrobial peptides, was a non-competitive and reversible inhibitor of 20S proteasome, and inhibited inflammation [41,42]. Our results are consistent with that report. pBD2 downregulated DEGs related to ribosome, oxidative phosphorylation, and proteasome, indicating that pBD2 could inhibit the occurrence of cancer, which needs further research. Some reported defensins have been regarded as a potential therapeutic target for cancer treatment, and attractive novel therapeutic candidates for antimicrobial and anticancer purposes [43,44], which is also consistent with our results.

pBD2 changed the transcriptional response caused by *E. coli*. There were 85 genes with reverse expression in the two DEGs group; KEGG analysis showed that these overlapping genes included enrichment of the microRNA in cancer (Figure 9). In addition, PTEN and CDC6 were key genes for pBD2 according to PPI net analysis (Figure 10). It has been reported that defensin regulated the expression of microRNA, and that the expression of miR-34a-5p increased in ethanol-induced liver injury in transgenic (TG) mice with overexpressing human neutrophil peptide 1, compared with WT (wild type) mice [45]. Those results perhaps imply that pBD2 had the ability to regulate gene expression by regulating microRNA, which needs further study. The genes associated with microRNA in cancer included PTEN, CDC25A, SPY2, PLAU, E2F2, and PTGS2. It was reported that PTEN could inhibit immune response and tumors [46]. SPRY2 was upregulated in glioblastoma, and overexpression of SPRY2 is associated with human oral squamous-cell carcinogenesis [47,48]. Dysregulation of PLAU is often accompanied by various cancers, and inhibition of PLAU expression could suppress tumor growth [49]. CDC25A, E2F2, and PTGS2 are all related to the cell cycle, and their dysregulation is related to tumors [50,51,52]. In this study, pBD2 showed anti-tumor potential by upregulating the expression of PTEN and downregulating the expression of SPRY2 and PLAU, and pBD2 was also shown to promote cell proliferation by upregulating the expression of CDC25A, E2F2, and PTGS2, which perhaps might lead to tumorigenesis. Some scholars have reported that defensin had a strong inhibitory effect on cancer [53,54,55], but others had opposite view suggesting that defensin might be related to the occurrence of cancer because the expression of defensins increased abnormally in cancer [56,57]. In addition to CDC25A, E2F2, and PTGS2 relating to the cell cycle, these genes are all related to cell proliferation including CDC6, CDK18, E2F8, CLSPN, CDCA7, MCM10, TIMELESS, KIF18B, etc., as mentioned above (Figure 10). These genes were all decreased in the *E. coli* group, and all increased by pBD2, indicating that pBD2 regulated cell proliferation and inhibited apoptosis, playing its role in the resolution of injury, infection, and inflammation [2]. pBD3 enhanced ovarian granulosa cell proliferation and migration [58]. Human β-defensins also stimulated various cellular activities, including keratinocyte proliferation, migration, and wound healing [59]. Those results are consistent with ours, which perhaps indicate that pBD2 could inhibit the occurrence of cancer, and promote cell proliferation to protect cells from infection. The analysis of the overlapping genes with reverse expression was consistent with the above analysis of DEGs in the *E. coli* and *E. coli* +pBD2 groups. Our results provide new insights into the potential function and mechanism of pBD2 against *E. coli,* and the results should be confirmed by further study on mice or human cell lines.

## 4. Materials and Methods

### 4.1. Strains and Cells

*E. coli* ATCC 25922 was purchased from the Beijing Ordinary Microbiology Strain Store Center (Beijing, China). The IPEC-J2 cells were a gift from Zhanyong Wei at the College of Veterinary Medicine, Henan Agricultural University.

### 4.2. Preparation for pBD2

pBD2 was expressed and purified in our laboratory by affinity chromatography based on the constructed strains BL21(DE3) pLysS-pET30a-*pBD2* [9]. The expression products were analyzed by SDS-PAGE, and the protein concentration was determined by bicinchoninic acid (BCA) assay (CW Biotech, Beijing, China).

### 4.3. Bactericidal Activity

The bactericidal activity was analyzed by scanning electron microscopy, as described previously [6]. *E. coli* ATCC 25922 was cultured in Luria Bertani (LB) medium until logarithmic growth stage at 220 rpm, then *E. coli* was incubated with 20 μg/mL pBD2 for 1 h or 4 h, and harvested by centrifugation. After being washed for 3 × 10 min in 10 mM PBS buffer, *E. coli* was fixed with 2.5% glutaraldehyde for 4 h, then dehydrated with gradient concentrations of ethanol, and the samples were observed by scanning electron microscopy (FEI Quanta 250, FEI, Hillsboro, OR, USA).

### 4.4. Cell Culture and Treatment

The IPEC-J2 cells were grown in 1640 medium supplemented with 10% fetal bovine serum (TianHang Biotechnology, Zhejiang, China) and 1% penicillin/streptomycin at 37 °C in an atmosphere of 5% CO_2_. The cells were seeded into a six-well plate and cultured until reaching 80% confluence, then at the logarithmic period (MOI = 50:1) the IPEC-J2 cells were challenged with *E. coli* or *E. coli* and 20 μg/mL pBD2 for 2 h in 1640 medium without fetal bovine serum and 1% penicillin/streptomycin. After washing with PBS three times, the cells were cultured for an additional 2 h in the medium without fetal bovine serum and 1% penicillin/streptomycin. After washing three times with PBS, 1 mL of TRIzon was added to the six-well plate, and the cells were lysed by pipetting, then the solute was transferred to RNase-free Eppendorf tubes for RNA extraction.

### 4.5. Library Preparation and Quality Control

RNAs were extracted using TRIzol reagents (CW Biotech, Beijing, China). The extracted RNAs were quantified using a spectrophotometer (Nanodrop 2000, Thermo Fisher Scientific, Waltham, MA, USA). A total of 3 μg RNA was sent to Biomarker Co., Ltd. (Beijing, China) to construct the cDNA libraries. Sequencing was performed on an Illumina HiSeqTM 2500 platform. The original raw data were saved in FASTQ file format. Each sequenced sample included two FASTQ files, containing reads from either end of the cDNA fragments. Quality control was performed to remove adaptor sequences, low-quality, and contaminated reads. Then the clean reads were mapped to the reference genome by alignment software HISAT2.

### 4.6. Analysis of DEGs

The transcript and gene expression levels were measured by FPKM (fragments per kilobase of transcript per million fragments mapped). The DESeq2 R package was used to analyze differential gene expression. The false discovery rate (FDR) was corrected using the Benjamini–Hochberg procedure. The threshold of FDR < 0.05 was used to filter out differential expressed genes (DEGs). Kyoto Encyclopedia of Genes and Genomes (KEGG) enrichment analysis of the DEGs were performed using BMKCloud (www.biocloud.net, accessed on 5 September 2019), based on the KEGG database (http://www.genome.jp/kegg/, accessed on 5 September 2019), and protein–protein interaction (PPI) networks of the DEGs were constructed on https://www.string-db.org/, accessed on 17 August 2021.

### 4.7. qRT-PCR

The extracted RNAs were converted to complimentary (c) DNA by a reverse transcriptase synthesis kit (DRR047A; TaKaRa Biotechnology, Shiga, Japan). The primers were designed using Primer Premier™ 5.0 (Sigma–Aldrich, Saint Louis, MO, USA) and are detailed in Table 4. Assays were performed with SYBR green dye (QIAGEN, Dusseldorf, Germany) using a real-time PCR cycler (LightCycler 96, Roche, Basel, Switzerland). The program was 95 °C for 30 s, 95 °C for 15 s, 60 °C for 30 s, 72 °C for 15 s, for 40 cycles. The results were analyzed using the 2^−ΔΔCT^ method with TUBA1B (tubulin alpha 1b) as a reference gene [60].

### 4.8. Statistical Analysis

All experiments were conducted with three biological replicates. Data were assessed by analysis of variance using SPSS Statistics 24 (IBM, Armonk, NY, USA).

## 5. Conclusions

In this study, IPEC-J2 cells were challenged with *E. coli*, and the effect of pBD2 on the gene expression of cells infected by *E. coli* was detected by transcriptome analyses. KEGG enrichment analysis revealed that DEGs between the control and *E. coli* groups were involved in signaling pathways related to immune responses. The DEGs between the *E. coli* and *E. coli* +pBD2 groups were involved in the ribosome, oxidative phosphorylation, and some disease pathways. These results indicate that pBD2 can play an active role in many biological processes including cancer, cell proliferation, and immunity. Furthermore, analysis of the overlapping genes with reverse expression in two DEG groups further revealed that pBD2 was involved in multiple biological processes to protect cells from *E. coli*. Our results provide new insights into the potential function and mechanism of pBD2 against *E. coli*. Meanwhile, this study provides a certain theoretical basis for the research and development of novel peptide drugs.

## Figures and Tables

**Table 1:** Sequencing data statistics.

Samples	Clean Reads	Clean Bases	GC Content	%≥Q30
Control-1	35,369,943	10,533,069,694	52.27%	94.10%
Control-2	32,617,012	9,703,386,646	52.26%	94.14%
Control-3	31,534,327	9,375,721,120	52.35%	94.31%
*E. coli*-1	24,055,348	7,170,857,754	52.64%	93.23%
*E. coli*-2	25,929,754	7,718,143,996	52.37%	93.55%
*E. coli*-3	22,754,029	6,773,668,654	52.28%	93.90%
pBD2-1	20,721,673	6,176,881,268	51.76%	93.46%
pBD2-2	23,894,463	7,107,703,946	52.28%	94.16%
pBD2-3	21,228,755	6,327,976,062	51.94%	93.67%

Notes: Control, *E. coli* and pBD2 indicate the IPEC-J2 in the control, *E. coli* and *E. coli* +pBD2 groups, and 1–3 indicate the three replicates, respectively.

**Table 2:** Gene comparison efficiency statistics.

Samples	Mapped Reads	Unique Mapped Reads	Multiple Map Reads
Control-1	67,898,715 (95.98%)	66,065,506 (93.39%)	1,833,209 (2.59%)
Control-2	62,849,152 (96.34%)	61,097,600 (93.66%)	1,751,552 (2.69%)
Control-3	60,714,671 (96.27%)	59,081,017 (93.68%)	1,633,654 (2.59%)
*E. coli*-1	46,168,875 (95.96%)	44,891,836 (93.31%)	1,277,039 (2.65%)
*E. coli*-2	49,843,598 (96.11%)	48,508,765 (93.54%)	1,334,833 (2.57%)
*E. coli*-3	43,776,016 (96.19%)	42,624,022 (93.66%)	1,151,994 (2.53%)
pBD2-1	39,790,781 (96.01%)	38,760,294 (93.53%)	1,030,487 (2.49%)
pBD2-2	46,057,704 (96.38%)	44,834,945 (93.82%)	1,222,759 (2.56%)
pBD2-3	40,692,378 (95.84%)	39,483,201 (92.99%)	1,209,177 (2.85%)

Notes: Control, *E. coli* and pBD2 indicate the IPEC-J2 in the control, *E. coli* and *E. coli* +pBD2 groups, and 1–3 indicate the three replicates, respectively.

**Table 3:** DEGs involved in the immune signaling pathways caused by *E. coli*.

KEGG Pathway	ID	Gene Name	Corrected *p*-Value
NF-kappa B signaling pathway	ko04064	TNFAIP3, BIRC3, CXCL8, TICAM1, NFKBIA, ICAM1, PTGS2, BCL10, PLAU, NFKB1, RELB, NFKB2	0.00000127
TNF signaling pathway	ko04668	TNFAIP3, MAP3K8, BIRC3, EDN1, NFKBIA, ICAM1, PTGS2, ENSSSCG00000008954, CXCL2, NFKB1, CCL20, BCL3	0.00000233
NOD-like receptor signaling pathway	ko04621	TNFAIP3, BIRC3, CXCL8, NFKBIA, NFKBIB, NFKB1	0.014302364
Chemokine signaling pathway	ko04062	FOXO3, RAC1, CXCL8, NFKBIA, NFKBIB, ENSSSCG00000008954, CXCL2, NFKB1, GNAI1, CCL20	0.014397446

**Table 4:** Primers used for qRT-PCR.

Genes	Sequence (5′ → 3′)		Size (bp)	GenBank Number
*MX1*	Forward	GTTACCGGGACAGCGAGATT	105	NM_214061.2
	Reverse	CATGACTGATTCCCACGCCT		
*EGFR*	Forward	AGGACGAAGCAACATGGTCA	132	NM_214007.1
	Reverse	TGCATAGCACAGGTTTCGGT		
*EIF2AK2*	Forward	CCCTGCACTTCTAGCCATCT	121	NM_214319.1
	Reverse	CGACCACTGGCCATTTCTTTC		
*PIK3R1*	Forward	CTTGAGTCGGGTGCTGGAAC	164	XM_021076847.1
	Reverse	AACGCGTCCCTAACCGATTC		
*PTEN*	Forward	TGCAATCCTCAGTTTGTGGT	224	NM_001143696.1
	Reverse	TCCTCTGGTCCTGGTATGAAG		
*OAS2*	Forward	AGCCAGAGCAATGGGAAACT	228	NM_001031796.1
	Reverse	GAGTTGCCCCTCAAGACTGT		
*ATP6VOB*	Forward	AACCCCAGCCTCTTCGTAAA	100	XM_021096834.1
	Reverse	TCACTCTGGAGGTCTGAAGG		
*RPL30*	Forward	GACAAGGTCCAATGTTCCCA	110	NM_001190178.1
	Reverse	CCAACCTCTTTTGTAGCCGT		
*NDUFA2*	Forward	TGCTAAGTGGCAAAGCCTG	167	XM_003124046.4
	Reverse	GGTAGAGGGTGGAACAAGGAA		
*NR4A1*	Forward	TGAGAAGGTTCCCGGCTTTG	196	ENSSSCG00000031321
	Reverse	GATGCTGTCGATCCAGTCCC		
*TUBA1B*	Forward	TACTCACCTCGACTCTTAGC	103	NM_001044544.1
	Reverse	GATGCACTCACGCATGG		

## Data Availability

All data generated during this study are included in this article. RNA-Seq data have been deposited in the National Center for Biotechnology Information database under accession number (PRJNA862330).
